# Impact of intraoperative medial collateral ligament injury on outcomes after total knee arthroplasty: a meta-analysis and systematic review

**DOI:** 10.1186/s13018-021-02824-5

**Published:** 2021-11-20

**Authors:** Jiahao Li, Zijian Yan, Yan Lv, Yijin Li, Pengcheng Ye, Peng Deng, Haitao Zhang, Jinlun Chen, Jie Li, Xinyu Qi, Jianchun Zeng, Yirong Zeng, Wenjun Feng

**Affiliations:** 1 The First Clinical Medical School, Guangzhou University of Chinese Medicine, Jichang Road 12#, District Baiyun, Guangzhou, Guangdong China; 2 The First Affiliated Hospital of Nanchang University, 17 Yongwai Street, Nanchang, 330006 China; 3 Department of Orthopaedics, The First Affiliated Hospital of Guangzhou University of Chinese Medicine, Jichang Road 16#, District Baiyun, Guangzhou, 510405 Guangdong China

**Keywords:** Total knee arthroplasty, Medial collateral ligament, Meta-analysis

## Abstract

**Background:**

As an uncommon but severe complication, medial collateral ligament (MCL) injury in total knee arthroplasty (TKA) may be significantly under-recognized. We aimed to determine whether MCL injury influences postoperative outcomes of patients undergoing TKA.

**Methods:**

Two independent reviewers searched PubMed, Cochrane Library, and EMBASE from their inception to July 1, 2021. The main outcomes were postoperative function, and secondary outcomes included the incidences of revision and complications.

**Results:**

A total of 403 articles yielded 15 studies eligible for inclusion with 10 studies used for meta-analysis. This study found that there was a statistically significant difference in postoperative functional scores, range of motion (ROM), complications, and revision rates, with adverse outcomes occurring more commonly in patients with MCL injury.

**Conclusions:**

This meta-analysis highlights the complexity of MCL injury during TKA and shows the impact on postoperative function, joint mobility, complications, and revision. Surgeons need to prevent and put more emphasis on MCL injury during TKA.

**Supplementary Information:**

The online version contains supplementary material available at 10.1186/s13018-021-02824-5.

## Background

As a well-established operation, total knee arthroplasty (TKA) was considered to be a highly effective method for the treatment of end-stage knee osteoarthritis [1]. Over the past decade, the number of total knee replacements performed annually has increased significantly. According to research, by 2030, the demand for primary total knee arthroplasty in the USA is expected to reach 3.48 million [2]. In this context, the increase in the revision rate may follow. Complications such as aseptic loosening, septic loosening, pain, and wear were the most common causes for revisions in TKA [3–5].

As an anatomical structure that restrains valgus and rotatory loads, the medial collateral ligament (MCL) is critical in providing stability after total knee arthroplasty [6, 7]. According to recent reports, the incidence of intraoperative injury to the MCL is about 0.5% to 3% [8–10], which includes transection injuries and avulsions of the femoral and tibial attachment [11–14]. It is possible for injury to occur during exposure of the knee and reduction after placement of prosthetic components [15]. In addition, the MCL can be damaged as a result of the direct injury caused by the saw blade and excessive release during surgery [16–18].

Based on the injury types, different treatment options can be adopted, including primary repair [9, 19, 20], augmentation with tendon graft [21–23], fixation with screws and washer construct [19], thicker polyethylene liner [14, 24], and the increase in prosthetic constraint [8, 10]. At present, a consensus has not yet been reached on the management of MCL injury during TKA, and the impact of the management on patients has remained undetermined. Hence, the purpose of this meta-analysis and systematic review was to review and summarize the available literature regarding MCL injury in TKA and evaluate whether MCL injury impacts clinical outcomes.

## Methods

### Search strategy

The conduction of this meta-analysis and systematic review followed the preferred reporting items for systematic review and meta-analysis (PRISMA) guideline. Subsequently, we searched the following databases: PubMed, Cochrane Library, and EMBASE, until July 1, 2021. To maximize the search results, our search strategy for these three databases followed Medical Subject Headings combination with terms (Additional file 1), but only included articles in English.

### Study selection and data extraction

All titles and abstracts were screened by two researchers (Zijian Yan and Yijin Li) using clearly defined inclusion and exclusion criteria. Only English-language publications on patients who reported MCL injuries during TKA were included for further examination.

According to the PICOS order, the study included in our meta-analysis had to meet all of the following requirements: (1) Population: patients undergoing primary total knee replacement; (2) Intervention: MCL injury group; (3) Comparison intervention: MCL-intact group; (3) At least one of the following indexes was assessed: functional outcomes, Knee Society Score, range of motion, postoperative pain score, complications, revision, and so on.

These studies will be excluded: revision knee replacement, biomechanics, physical and animal studies, conference abstracts, case reports, comments and reports of undefined MCL injuries.

Data extraction of all included studies was performed independently by two authors (Zijian Yan and Yijin Li) according to the Cochrane guidelines. Relevant data extracted included publication information (author, study design, and year) and patient baseline characteristics (gender, body mass index [BMI], age, and type of prosthesis). Injury type (transection or avulsion), outcome data, and management were also extracted.

### Quality assessment

Newcastle–Ottawa Scale (NOS) tool was used to assess methodological quality in any of the included studies [25]. This scale contains eight items, which are divided into three dimensions: selection, comparability, outcome measurement. All studies were independently evaluated by two researchers, and disagreements were resolved through discussion by a third reviewer.

### Statistical analysis

All extracted data analysis and picture production were performed with the Review Manager (version 5.4 for Windows). To evaluate the dichotomous variables in the study (such as postoperative complications), we commonly selected the odds ratio (OR) and the associated 95% confidence interval (CI) to measure. Given that the incidence is rare, the reported OR can be approximated as RR (relative risk) based on Cornfield’s research results [26]. Then, we included studies that provided complete mean and standard deviation. Mean difference (MD) or standard mean difference (SMD) were used to analyze continuous variables such as KSS or KFS. *I*^2^ and *Q* tests were used to evaluate the heterogeneity between studies. For heterogeneity testing, when *I*^2^ ≥ 50%, the random effects model was used to replace the fixed effects model [27]. The forest map was used to display the results of the aggregate effect size analysis of each study, while the Deeks’ funnel plot was applied to evaluate the publication bias.

## Results

### Study selection

**Fig. 1:**
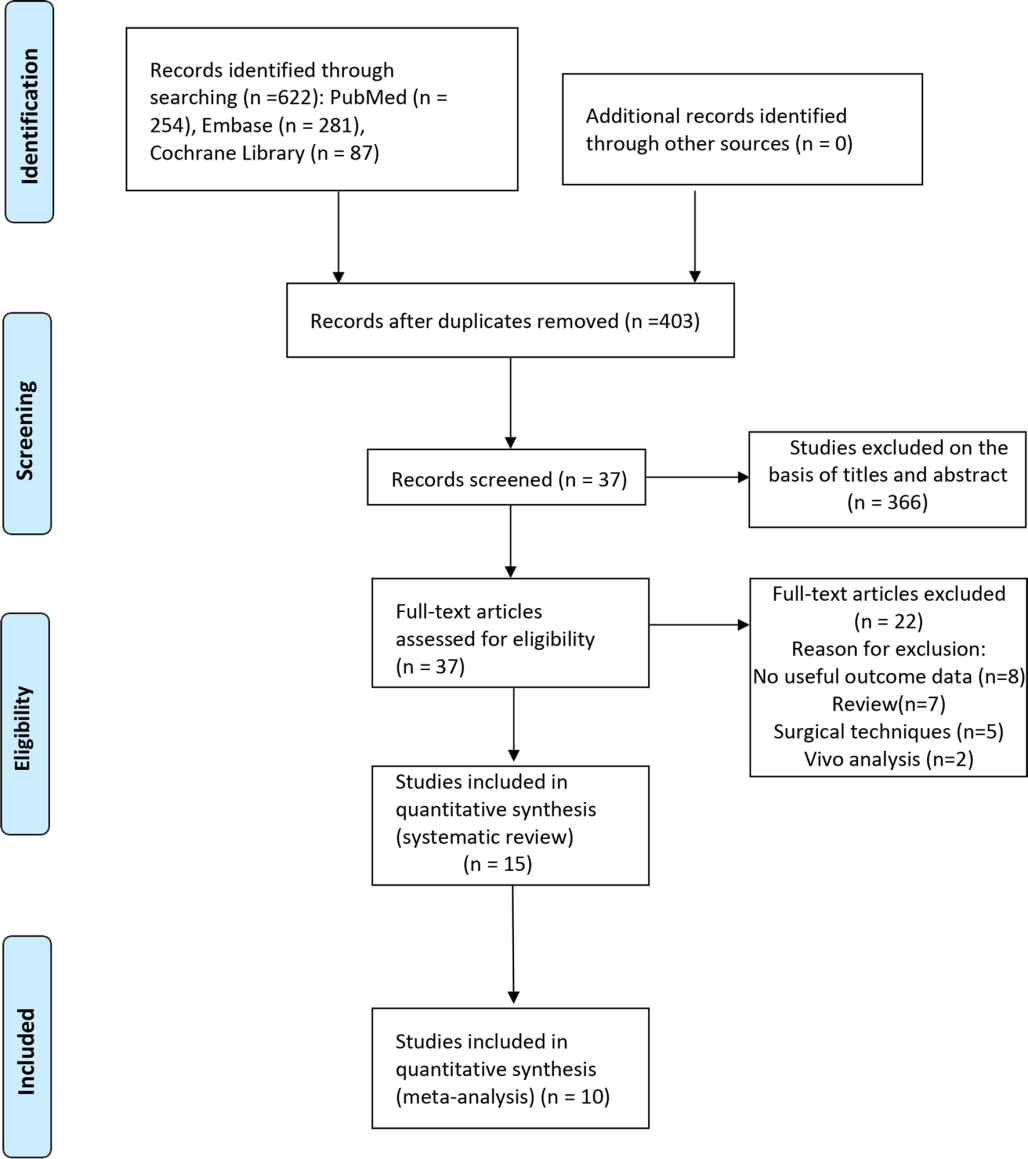
The search strategy flowchart of study selection

Following the search strategy described above, a total of 622 relevant papers were initially screened from the three databases. After deleting the duplicate literature, 403 articles remained. By reading the titles and abstracts, 366 studies that did not meet our requirements were removed, leaving 37 articles for further reading in full-text. Finally, 15 articles were included in the systematic review and 10 articles were included in the meta-analysis after reading the full-text, with reasons for exclusion included review, no available outcome data, surgical technique, and in vitro studies. The complete literature screening process was illustrated as PRISMA flow diagram in Fig. 1.

### Study characteristics and quality assessment

Demographics and clinical outcomes of the included studies were summarized in Tables 1 and 2. Among the 15 screened citations, nine were cohort studies [8, 10, 12, 13, 20, 24, 28–30], five were retrospective studies [9, 14, 19, 22, 31], and one was a case–control study [11]. A total of 376 knees in the medial collateral ligament injury group were studied in comparison with 5025 knees in the control group with intact medial collateral ligaments. Notably, 166 knees had an intraoperative injury with tear in the mid-substance, while the other 220 knees were avulsion injuries. In terms of clinical outcomes, 11 studies evaluated KSS scores, nine papers compared KFS scores, and six papers had documented ROM in their entirety. Complications and revisions were reported in 7 of the 15 studies, with common reasons such as stiffness, instability, and infection. The quality of 10 studies included in the meta-analysis assessed with the Newcastle–Ottawa scale, ranged from six to eight. Among them, three studies scored 6 points, five studies scored 7 points, and two study scored 8 points (Table 3).

**Table 1:** Demographics of the included studies

Author	Years	Design	Sample size*	Mean age*	BMI*	Follow-up (Mon)*	Outcome Measures
Leopold [9]	2001	Retrospective study	16 (2.6%)	63	32.5	45	Revision, HSS, ROM
Koo [24]	2009	Cohort study	15/11	63.9	NR	24	Revision, KSS, KFS, ROM
Lee [8]	2011	Cohort study	37/1613	60	NR	44	Revision, Complications, KSS, KFS
Dragosloveanu [14]	2013	Retrospective study	8 (1.8%)	62.8	34	12	Revision, Complications, KSS, KFS
Siqueir [10]	2014	Cohort study	23/92	66.5/69.1	32.7/32.8	60.3/52	Revision, KSS, KFS
Shahi [22]	2014	Retrospective study	15 (0.43%)	64	38	16	Revision, KSS, Coronal alignment
Cao [13]	2016	Cohort study	11/18	64.3/63.7	26.75/26.37	15.8/19.5	Revision, KSS, KFS
Bohl [19]	2016	Retrospective study	35 (1.2%)	62	34	99	Revision, Complications, HSS, ROM
Wang [12]	2017	Cohort study	17/1732	63/60.7	34.4/34.6	51	Revision, KSS, KFS
White [30]	2018	Cohort study	33/770	63.6/63.6	32.4/30.4	31.2	Revision, Complications, KOOS, VAS
Jin [20]	2019	Cohort study	65/65	71.4/69.2	26.4/26.2	74.1/79.8	Revision, KSS, WOMAC, ROM
Motififard [28]	2020	Cohort study	35/618	68/66	NR	24	Revision, Complications, KSS, KFS, ROM
Ni [31]	2020	Retrospective study	14	63.6	27.2	15.6	Revision, HSS, ROM, Coronal alignment
Rajkumar [11]	2020	Case–control study	41/82	65.2/64.6	33.8/33.9	58.4	Revision, Complications, KSS, KFS, ROM
Sun [29]	2020	Cohort study	11/24	64.2/63.5	28.33/27.47	35.5/36	Revision, KSS, KFS

*The values were given as the number with MCL injury/intact

KSS, Knee Society Score; KFS, Knee Society Functional Score; ROM, range of motion; KOOS, Knee Injury and Osteoarthritis Outcome Score; WOMAC, Western Ontario and McMaster Universities Osteoarthritis Index; VAS, visual analog scale; NR, not reported

**Table 2:** Summary of clinic outcomes for each study

Author	Years	MCL injury		Implant	Management	KSS*	KFS*	Complications and revision	ROM*
		Transection	Avulsion						
Leopold [9]	2001	12	4	12CR/4PS	Suture anchors/screw-and-washer/ suture repair	NR	NR	1 PJI (1 revision)	G1:108
Koo [24]	2009	0	15	13PS/2CR	Thicker polyethylene insert	G1:91 ± 6.78 / G2: 92.20 ± 3.74	G1:82.5 ± 13.57/ G2:82.00 ± 3.59	0	G1:130 ± 9 / G2: 130 ± 13
Lee [8]	2011	28	9	7PS/30 TCIII	14 ligament repair /23NR	G1:81/G2:91	G1:74/G2:87	4 instability/1 PJI/2 aseptic loosening (7 Revision)	NR
Dragosloveanu [14]	2013	1	7	5PS/3 constraint	7 suture anchor/1 suture repair	GI:87.7	G1:80	1 instability (1 revision)	NR
Siqueir [10]	2014	22	1	10PS/2CR/ 11constraint	10 ligament repair/2 unconstrained implant /11 constrained implant	G1:78.8 ± 24.4/ G2:86.7 ± 21	G1:67.8 ± 22.9/ G2:72.2 ± 25.2	0	NR
Shahi [22]	2014	11	4	NR	15 synthetic ligament	G1:92	NR	0	NR
Cao [13]	2016	10	1	8PS/3CR	11 ligament repair	G1:89.82 ± 3.76/ G2:90.19 ± 3.39	G1:89.54 ± 3.50/ G2:90 ± 3.53	0	NR
Bohl [19]	2016	24	21	10PS/35CR	Suture anchors/screw-and-washer/ suture repair	NR	NR	5 stiffness (1 revision), 2 aseptic loosening (2 revision)	G1:110
Wang [12]	2017	12	5	CR	Ligament reconstruction	G1:87.7 ± 6.2 / G2:90.6 ± 6.9	G1:84.7 ± 5.9 / G2:87.9 ± 7.6	0	NR
White [30]	2018	0	33	PS/CR	Using Bone Staples	NR	NR	6 subjective instability/4 moderate to severe instability	NR
Jin [20]	2019	0	65	PS	36 suture anchor/29 staple	G1:87.3 ± 7.3 / G2:87.6 ± 10.1	NR	0	G1:125.6 ± 8.9/ G2:128.1 ± 8.1
Motififard [28]	2020	35	0	PS	Nonabsorbable braided suture repair	G1:81 ± 17/ G2:86 ± 15	G1:61 ± 13/ G2:67 ± 5	5 coronal instability (3 Revision)	G1:100 ± 13/ G2:107 ± 8
Ni [31]	2020	0	14	10PS/2CR/2CCK	Screw-and-washer	NR	NR	0	G1:103.9 ± 6.8
Rajkumar [11]	2020	0	41	PS	Screw and washer construct fixation	G1:85(80 ~ 90)/ G2:85(81 ~ 85)	G1:90(80–95)/ G2:90(85–90)	1 screw back-out/ 1 debridement for hematom	NR
Sun [29]	2020	11	0	PS	Meniscus autograft transfer	G1:95 ± 4.47/ G2:95.4 ± 3.88	G1:91.8 ± 7.5/ G2:90.4 ± 7.5	0	NR

*The values were given as the number with MCL injury/intact

PS. posterior stabilized; CR, cruciate retaining; NR, not reported; NR, not reported

**Table 3:** Quality assessment for the studies included in the meta-analysis (NOS)

Study	Selection	Comparability	Exposure or outcome	Total score
Koo [24]	★★★	★★	★★	7
Lee [8]	★★	★	★★★	6
Siqueir [10]	★★★	★★	★★★	8
Cao [13]	★★★	★★	★★	7
Wang [12]	★★★	★★	★★	7
White [30]	★★	★★	★★	6
Jin [20]	★★	★	★★★	6
Motififard [28]	★★★	★★	★★★	8
Rajkumar [11]	★★	★★	★★★	7
Sun [29]	★★★	★	★★★	7

★★★ indicates strong level of evidence; ★★ indicates moderate level of evidence, ★ indicates limited level of evidence

*NOS*, Newcastle–Ottawa scale

### Knee Society Score (KSS)

**Fig. 2:**
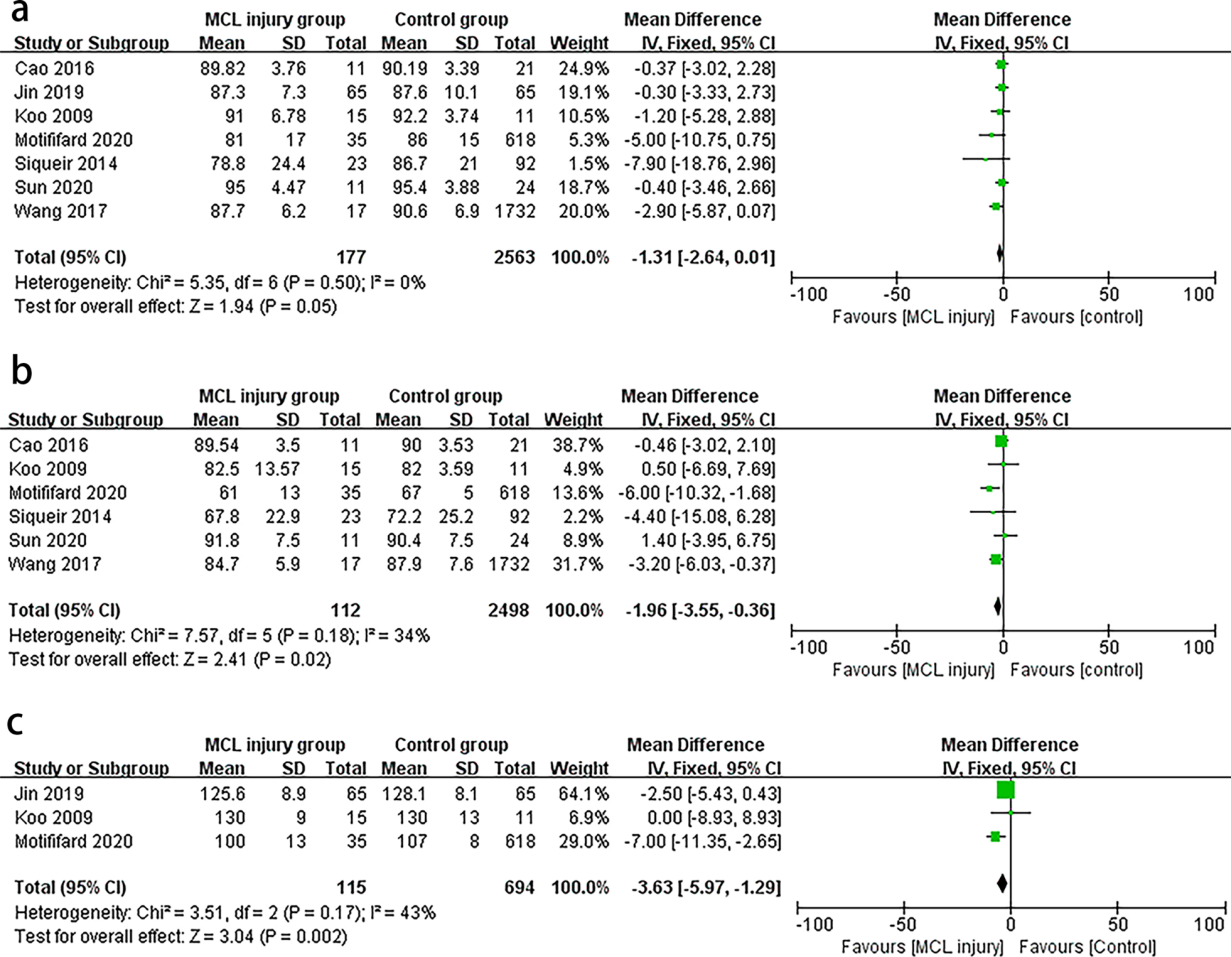
Forest plots for the KSS (**a**), KFS (**b**), and ROM (**c**). KSS, Knee Society Score; KFS, Knee Society Functional Score; ROM, range of motion; CI, confidence interval

**Fig. 3:**
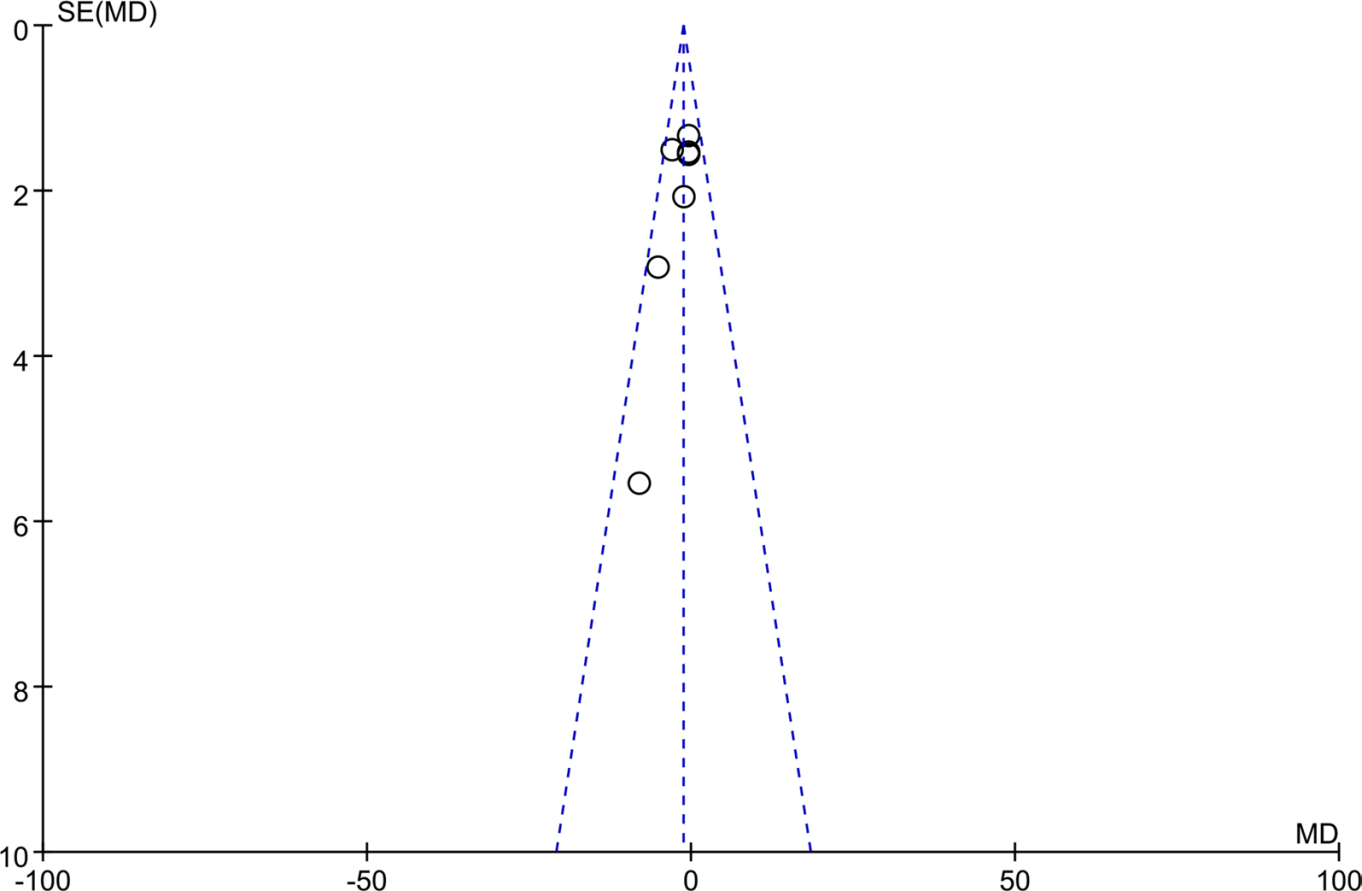
Funnel plots for reporting the KSS

The KSS score was used in nine studies [10, 12–14, 20, 22, 24, 28, 29] and the results in meta-analysis showed significant differences after MCL injury (MD − 1.31, 95% CI − 2.64 to 0.01, *P* = 0.5, *I*^2^ = 0%; Fig. 2a). In this meta-analysis, we chose a fixed effect model because the results of the heterogeneity analysis (*P* = 0.05, *I*^2^ = 0%) indicated essentially no heterogeneity. Sensitivity analysis showed no literature that would significantly interfere with the results of the analysis, representing good accuracy and stability of this study. The pooled information was shown in our forest plot (Fig. 2a), and the results revealed that intraoperative injury to the MCL during TKA significantly reduces the postoperative KSS score. To clarify whether publication bias exists, a funnel plot (Fig. 3) was generated to examine. In Fig. 3, the funnel plot appeared symmetrical, which indicated the absence of publication bias.

### Knee Function Score (KFS)

Six studies [10, 12, 13, 24, 28, 29] provided sufficient information and were included in this meta-analysis. Similarly, fixed effects models were used to calculate because no evidence of heterogeneity was found in the study (MD −1.96, 95% CI −3.55 to −0.36, *P* = 0.18, *I*^2^ = 34%). The pooled data showed that MCL injury also significantly decreased KFS scores compared to the control group (Fig. 2b).

### Range of motion (ROM)

ROM was reported in six articles, and three of them met the inclusion criteria [20, 24, 28]. Patients in the MCL injury group had worse mean postoperative ROM compared to those in the MCL-intact group (MD −3.63, 95% CI −5.97 to − 1.29, *P* = 0.17, *I*^2^ = 43%) (Fig. 2c).

### Complications and revision

**Fig. 4:**
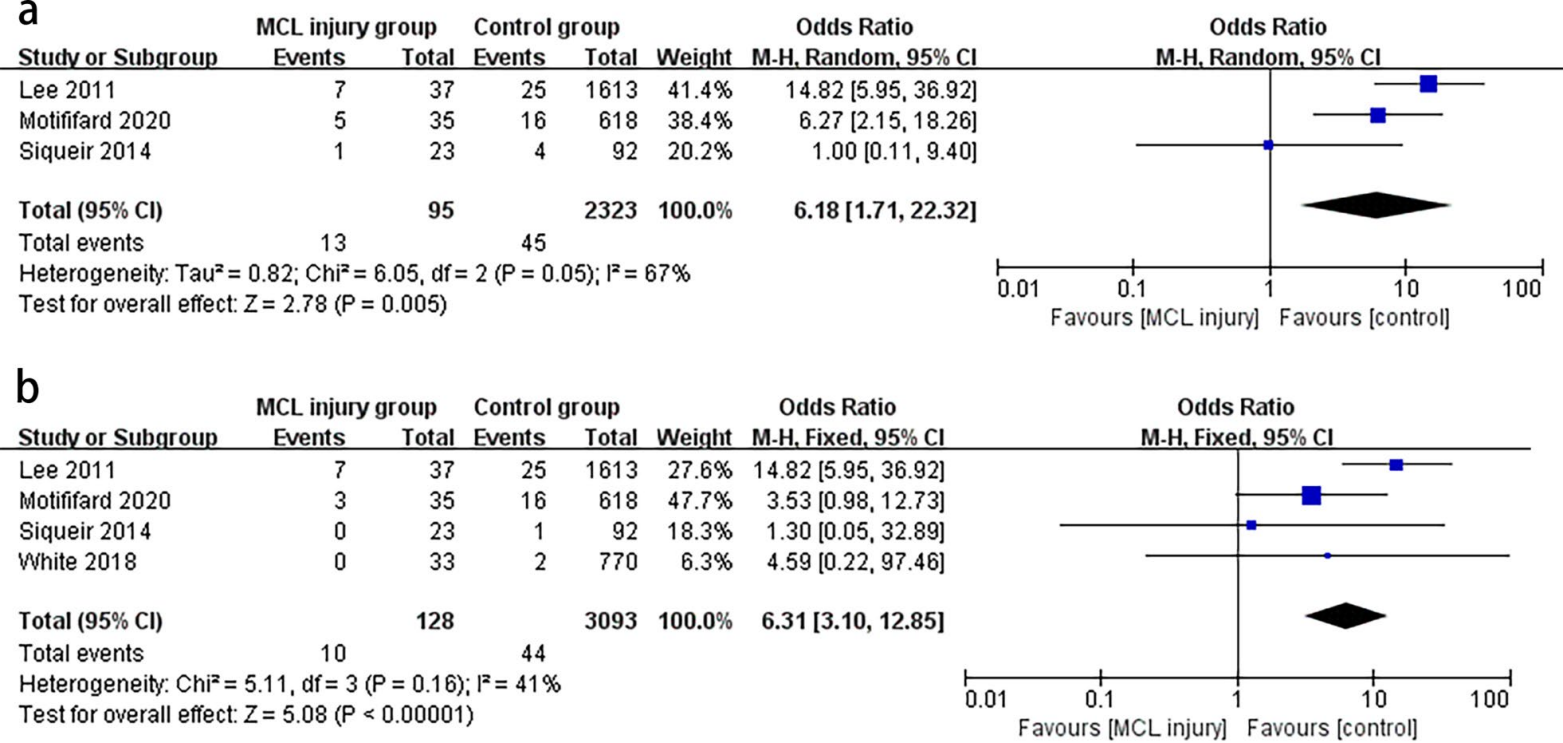
Forest plots of the complications (**a**) and revision (**b**) between MCL injury group and control group after primary TKA

After excluding studies without complications and revision, four [8, 10, 28, 30] and three studies [8, 10, 28] were pooled into the analysis of complications and revisions, respectively. According to Fig. 4, the complication (MD 6.18, 95% CI 1.71 to 22.32, *P* = 0.05, *I*^2^ = 67%; Fig. 4a) and revision rates (MD 6.31, 95% CI 3.10 to 12.85, *P* = 0.16, *I*^2^ = 41%; Fig. 4b) were six folds higher in the MCL injury group than in the control group. Lee et al. reported seven complications including four instabilities, two aseptic loosening, and one PJI, all of which were eventually revised to TCIII prostheses using cemented femoral and tibial stems [8]. In the study by Motififard et al. [28], five patients treated for MCL insufficiency developed coronal instability, three of whom undergone revision. Furthermore, complications such as instability, screw loosening, and postoperative hematoma were reported in the study by Rajkumar and White, which were no clear indications of revision [11, 30].

## Discussion

As an uncommon but severe complication, MCL injury in total knee arthroplasty may be significantly under-recognized. Avulsion damage to the MCL, or transection in the middle, can lead to poor postoperative function, instability, loosening, and accelerated polyethylene wear [15]. This was confirmed in our study. This systematic literature review and meta-analysis aimed to report the impact of intraoperative MCL ligament injury on patients undergoing TKA, which may provide recommendations for orthopedic surgeons regarding the treatment of MCL injury. This meta-analysis included 10 studies (9 cohort trials and 1 case–control trial) that analyzed 5313 knees and directly compared the clinical outcomes of the MCL-injured group with those of the MCL-intact control group. Pooled data showed significant differences between the two groups in terms of KSS, KFS, ROM, complications and revision rates. On the basis of the available evidence, injury to the MCL during total knee arthroplasty significantly affects surgical outcomes.

The reasons for MCL injury in TKA are complex and multi-factorial. Some of them are avoidable iatrogenic injury by careful preoperative history-taking and physical examination, and the other part depends on the surgeon’s intraoperative operation. According to our aggregated data, avulsion injuries account for most injuries (59%), followed by mid-substance disruptions (41%) [8, 10–13, 20, 24, 28–30]. MCL injuries are most common in medial soft tissue release or hyperflexion of the knee during subluxation of the tibia or while trial components were placed in a tight flexion gap [15]. In Rajkumar et al. [11] series, severe varus deformity, knee subluxation and “cup and saucer” shape before surgery were risk factors for MCL avulsion injury. In some cases, due to insufficient protection by retractors, the saw blades that cut the bone can cause direct trauma of the ligament [16, 32]. Finally, morbid obesity was also a risk factor for injury, Winiarsky et al. [33]reported 4 cases of intraoperative MCL avulsion injury among 50 morbidly obese patients (8%), which was significantly higher than that in the control group.

There was no consensus on the optimal management of intraoperative MCL injuries, but the aim was to reconstruct the medial–lateral balance of the knee and maintain coronal stability [34]. Most scholars had addressed this problem by using constrained implants that can restore stability to the knee joint after surgery [8, 10, 14]. However, the application of constrained implants may increase the stress on the bone cement and prosthesis-bone interface, and the accompanying greater bone loss can make revision difficult [35]. Previous findings had shown that the medial collateral ligament had a good ability to heal after injury [36–38]. Therefore, some scholars adopted for a conservative approach and reported good clinical results [10, 24, 39]. However, it should be applied with caution to patients with high activity requirements [37]. Currently, primary repair of the MCL was usually in the form of suture repair for the disruption of transection and suture anchor or screw-and-washer reattachment for avulsion of the collateral ligament from the femoral or tibial attachments [9, 19, 20, 30]. Meanwhile, reconstruction of the MCL has been advocated to treat intraoperative MCL injuries, including the use of autologous quadriceps tendon [21], semitendinosus tendon [12], thin femoral tendon [13], and artificial ligaments [22].

The reasons for the lower scores in patients with MCL injuries in TKA have not been elucidated clearly, but are likely due to instability and stiffness of the knee. Our meta-analysis also showed that the revision rate was higher in the repaired group than in the control group. Of these, only two cases of infection were reported in the study by Lee et al. [8] and Leopold et al. [9]. Therefore, non-infectious complications such as aseptic loosening or instability are regarded as the primary cause for revision after TKA due to its frequency and severity. Traditionally, superficial MCL (sMCL) and deep MCL (dMCL) were important anatomical structures for maintaining knee stability, especially in limiting internal and external rotation [40–42]. In our study, a total of 24 patients reported postoperative instability and aseptic loosening, and 12 patients eventually required revision [8, 14, 19, 28, 30]. Notably, the study by White et al. [30] used bone staples to treat superficial MCL injuries and reported 10 instances of instability (30%). The incidence was significantly higher than other studies, which we believe was related to the use of an independent questionnaire for assessing stability [30]. Similarly, in the study by Motififard et al. [28], the postoperative instability rate in the MCL repaired group was notable. They attributed this to the use of the pie‑crusting technique in the varus deformity. Poorer Postoperative score may result from the stiffness in the repaired group, which may inhibit the range of motion and therefore, patient-reported function. More than 10% of patients required intervention for stiffness from the report by Bohl et al. [19], and they considered that it may be associated with the use of the hinged knee brace. This finding indicates that when using a hinged knee brace, more emphasis should be placed on the exercise of the range of motion.

This systematic review and meta-analysis are the first to be conducted on MCL injury and clinical outcomes after TKA. However, this study still has its own limitations. Firstly, there is complexity in the spectrum of MCL injury and factors affecting ligament healing, and it has not been reported in detail, so there is heterogeneity among included studies. We tried to contact the authors to obtain the original data, but failed due to time constraints. Therefore, we cannot perform a subgroup analysis to see if the functional outcomes were different with studies reporting avulsions versus mid-substance transections. Secondly, most of the included studies are retrospective cohort studies, which represents that the level of evidence is moderate, and the reliability of the findings needs to be confirmed. Thirdly, MCL injury is a rare complication and the studies we included showed few cases of adverse outcomes and revisions, so longer follow-up and more studies are needed to prove the conclusions of our study.

## Conclusion

Patients receiving TKA with intraoperative MCL injury are at an increased risk of complications and revision in comparison to patients without. Poorer functional outcomes are also associated with MCL injury, although further clarification in future studies is required. It is recommended that surgeons are expected to pay particular attention to these patients, and improve preoperative preparation and surgical techniques to prevent intraoperative MCL injury.

## Supplementary Information

**Additional file 1.** Detailed search strategy of Pubmed.

## Data Availability

The authors declare that all the data supporting the findings of this study are available within the article and its supplementary information files.

## Abbreviations

TKA: Total knee arthroplasty
MCL: Medial collateral ligament
ROM: Range of motion
KSS: Knee Society Score
KFS: Knee Function Score
